# Photosensitivity reaction induced by finasteride

**DOI:** 10.1002/jgf2.510

**Published:** 2021-11-05

**Authors:** Takayuki Yamada

**Affiliations:** ^1^ Asunaro Clinic Takasaki City Japan

**Keywords:** finasteride, photosensitivity reaction

## Abstract

The patient visited our office complaining of a desquamating eruption on his face since two months. On extensive history‐taking, he had received oral finasteride for four months to prevent alopecia androgenetica, indicating drug‐induced photosensitivity. Withdrawal of finasteride for two weeks resulted in an improvement in the facial rash.

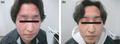

A 34‐year‐old man visited our office complaining of a desquamating eruption on his face since two months. During the two months, he visited two independent dermatologists who diagnosed the lesion as seborrheic eczema and miliaria, respectively, and prescribed steroid ointment treatment, which was ineffective. The rash on his face revealed scaling erythematous macules (Figure 1A). Additionally, both dorsal surfaces of the hands indicated sun exposure effects. After extensive history‐taking, it was noted that he had received oral finasteride for four months to prevent alopecia androgenetica, thus indicating drug‐induced photosensitivity. Withdrawal of finasteride for two weeks resulted in an improvement in the facial rash (Figure 1B).

**FIGURE 1:**
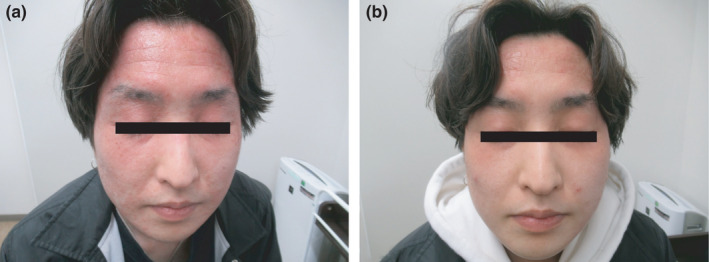
(A) Rash on the face revealing scaling erythematous macules. (B) Withdrawal of finasteride for 2 weeks resulting in improvement of the face rash

Dermatologists often use a snapshot diagnosis to make a decision based on experience to continue clinical practice. This could be because of the lower medical and technical service compensation when compared to the average physician fee in Japan,^1^ resulting in a relatively shorter average time spent with a new patient.

Diseases causing facial erythema include rosacea, seborrheic dermatitis, atopic dermatitis, psoriasis, contact dermatitis, photosensitive disorders including sunburn, polymorphous light eruption, or phototoxic and photoallergic eruption, systemic disorders including lupus erythematous and dermatomyositis, parvovirus B19 infection, and erysipelas.^2^

Scaly face diseases include eczema, contact dermatitis, some forms of collagen disease, and drug‐induced photosensitivity. Contact dermatitis induced by eczema and cosmetics is quite frequent in the primary setting. Inadequate drug history‐taking may cause a missed diagnosis of drug‐induced photosensitivity. However, detailed history‐taking and physical examination prove to be a challenge in all outpatients, considering the limited amount of time. Additionally, the patient did not recall the potential relevance of finasteride taken orally for two months, and hence did not report it. Physicians must elicit information regarding drug use to recognize this drug‐induced photosensitivity phenomenon under these conditions.

Drug photosensitivity is an abnormal skin reaction in individuals on known causative drugs who are exposed to light, usually ultraviolet light, because of the presence of endogenous or exogenous chromophores in the dermis or epidermis. Drug photosensitivity can present in a wide spectrum of acute or delayed clinical patterns. It is important that these be identified and distinguished from idiopathic photodermatoses, in order to remove the offending drug and/or take adequate measures to reduce this adverse effect.^3^ Various orally administered drugs, including finasteride, may cause erythema.^4^ However, while our clinic has prescribed finasteride for many patients, we report here our first experience with a photosensitivity reaction induced by finasteride. A definitive diagnostic procedure would be a challenge test of oral intake of finasteride. However, the patient's lesion almost completely healed, and a challenge test was unnecessary.

## CONFLICT OF INTEREST

The authors have stated explicitly that there are no conflicts of interest in connection with this article.

## INFORMED CONSENT

Informed consent was obtained from the patient for the publication of case details.
